# Characterization of deltamethrin degradation and metabolic pathway by co-culture of *Acinetobacter junii* LH-1-1 and *Klebsiella pneumoniae* BPBA052

**DOI:** 10.1186/s13568-020-01043-1

**Published:** 2020-06-03

**Authors:** Jie Tang, Qiong Hu, Dan Lei, Min Wu, Chaoyi Zeng, Qing Zhang

**Affiliations:** Key Laboratory of Food Biotechnology, School of Food and Biotechnology, Xihua University, Chengdu, 610039 Sichuan People’s Republic of China

**Keywords:** Deltamethrin, 3-phenoxybenzoic acid, Co-culture, *Acinetobacter junii* LH-1-1, *Klebsiella pneumoniae* BPBA052

## Abstract

Deltamethrin and its major metabolite 3‐phenoxybenzoic acid (3‐PBA) have caused serious threat to the environment as well as human health, yet little is known about their degradation pathways by bacterial co-cultures. In this study, the growth and degradation kinetics of *Acinetobacter junii* LH-1-1 and *Klebsiella pneumoniae* BPBA052 during deltamethrin and 3-PBA degradation were established, respectively. When the inoculum proportion of the strains LH-1-1 and BPBA052 was 7.5:2.5, and LH-1-1 was inoculated 24 h before inoculation of strain BPBA052, 94.25% deltamethrin was degraded and 9.16 mg/L of 3-PBA remained within 72 h, which was 20.36% higher and 10.25 mg/L lesser than that in monoculture of LH-1-1, respectively. And the half-life of deltamethrin was shortened from 38.40 h to 24.58 h. Based on gas chromatography–mass spectrometry, 3-phenoxybenzaldehyde, 1,2-benzenedicarboxylic butyl dacyl ester, and phenol were identified as metabolites during deltamethrin degradation in co-culture. This is the first time that a co-culture degradation pathway of deltamethrin has been proposed based on these identified metabolites. Bioremediation of deltamethrin-contaminated soils with co-culture of strains LH-1-1 and BPBA052 significantly enhanced deltamethrin degradation and 3-PBA removal. This study provides a platform for further studies on deltamethrin and 3-PBA biodegradation mechanism in co-culture, and it also proposes a promising approach for efficient bioremediation of environment contaminated by pyrethroid pesticides and their associated metabolites.

## Keypoints

- Co-culture was used to degrade both deltamethrin and its toxic intermediate metabolite 3-PBA.
- Co-culture was more efficient in degradation of deltamethrin and 3-PBA than the monoculture.
- A degradation pathway in co-degradation of deltamethrin and 3-PBA was first reported.
- Deltamethrin-contaminated soils could be efficiently bioremediated by co-culture.

## Introduction

Pyrethroid pesticides (PPs) are a type of synthetic insecticides, which are widely used in agriculture, forestry, horticulture, public health, and indoor places to control pests, cockroaches, mosquitoes, and livestock parasites because of their broad spectrum, high efficiency, and low mammalian toxicity as compared to that of organophosphate pesticides (Cycoń and Piotrowska-Seget 2016; Zhan et al. 2018). Deltamethrin, belongs to type II PPs, which has high insecticidal activity and has been commonly and widely used against insect pests (Hao et al. 2018). However, its continuous and extensive using not only resulted in serious contamination of the environment, but also negatively affected the non-target organisms (Li et al. 2008), as it shows neurotoxicity, reproductive toxicity, immunotoxicity, and acts as an endocrine disruptor (Zhang et al. 2010; Brander et al. 2016). Recently, deltamethrin has received increasing attention because of its major metabolite, 3-phenoxybenzoic acid (3-PBA) (Hao et al. 2018; Braganca et al. 2019; Zhang et al. 2016). Many studies have suggested that 3-PBA with stronger hydrophilicity is more likely to spread in the environment than its parent compounds, making it difficult to degrade and easy to accumulate (Chen et al. 2012b). Moreover, 3-PBA is a toxic and persistent hydrophobic compound, and its antibacterial activity is a major obstacle for microbial growth and further degradation of its parent compound (Chen et al. 2011b, 2012b; Hu et al. 2018). Meanwhile, it is considered as an endocrine disruptor due to its antiestrogenic activity (Chen et al. 2011b, 2012a). Therefore, effective measures for simultaneous degrading and removal of deltamethrin and its intermediate metabolite 3-PBA are crucial to ensure that human and environmental health is not influenced by the excessive use of deltamethrin.

Currently, biodegradation has become a mainstream technology for removal of pesticide residues or pesticide metabolites, as it is eco-friendly, cost-effective, convenient, and possesses excellent degradation properties (Hao et al. 2018). At present, a variety of microorganisms have been used to degrade pesticides or their metabolites, such as *Bacillus* sp. (Liu et al. 2015; Chen et al. 2015; Tang et al. 2018b), *Pseudomona*s sp. (Halden et al. 1999), *Sphingomonas* sp. (Tang et al. 2013), *Stenotrophomonas* sp. (Chen et al. 2011c), and *Aspergillus* sp. (Zhu et al. 2016; Deng et al. 2015). However, most of these microorganisms cannot completely mineralize pesticides or their metabolites alone (Chen et al. 2012b). Zhao et al. (2016) reported that about 50% β-cypermethrin (100 mg/L) could be degraded by *Bacillus licheniformis* B-1 after 72 h, but was unable to sustain mineralization of 3-PBA; Zhu et al. (2016) reported that 80.62% 3-PBA (100 mg/L) could be degraded by *Aspergillus oryzae* M-4 within 5 d, but did not clear its ability to degrade PPs. Therefore, a new idea emerged, wherein synergy between strains to simultaneously degrade pyrethroid pesticides and their toxic intermediate metabolites (Jones and Wang 2018). Compared with other biodegradation methods, in co-cultures, microbes are more adaptable to the environment and can achieve more rapid pathway optimization, thereby improving the efficiency of pollutant degradation (Zhao et al. 2016; Jones and Wang 2018). Recently, some reports have verified that microbial co-cultures can effectively degrade organic pollutants and their related metabolites (Zhao et al. 2016; Tran et al. 2013). Liu et al. (2014) studied the co-degradation of cypermethrin and 3-PBA by strains *Bacillus licheniformis* B-1 and *Sphingomonas* sp. SC-1, the degradation rate of cypermethrin (100 mg/L) was 75.60% in 72 h and the content of 3-PBA was 10.31 mg/L. As compared with the degradation by strain B-1 alone, the degradation of cypermethrin increased by 24.52% and 3-PBA content reduced by 12.5 mg/L in case of degradation of cypermethrin in co-culture. However, co-culture pathways for degradation of deltamethrin and its intermediate metabolites have remained largely unexplored.

In this study, co-culture of *Acinetobacter junii* LH-1-1 and *Klebsiella pneumoniae* BPBA052 was used for simultaneous degradation of deltamethrin and its toxic intermediate metabolite 3-PBA. The growth and degradation kinetics, inoculum ratio, inoculation sequence, and soil bioremediation ability of the co-culture strains were studied. In addition, this report contains the first proposal of a microbial co-culture degradation pathway for degrading deltamethrin and 3-PBA based on metabolite identification. This study also provided a promising approach for further bioremediation environment contaminated by PPs or 3-PBA.

## Materials and methods

### Chemicals and culture conditions

Deltamethrin (98.5%) was purchased from Ronch Chemicals (Nanjing, China), 3-PBA standard (98.0%) and chromatographic-grade acetonitrile were obtained from TCI (Shanghai, China) and Adamas-Beta (Shanghai, China), respectively. Other chemicals were analytical grade and commercially available.

The liquid medium used for degradation of deltamethrin by the bacteria was optimized mineral salt medium (OMSM), containing 0.5 g/L KH_2_PO_4_, 1.5 g/L K_2_HPO_4_, 1.5 g/L (NH_4_)_2_SO_4_, 0.2 g/L MgSO_4_, 0.5 g/L NaCl, 0.01 g/L FeCl_3_, and supplemented with 3 g/L tryptone as extra carbon source, the pH was adjusted to 7.0. At each stage, pre-cultures were incubated in Luria–Bertani (Tang et al. 2018a) (LB) liquid medium for 24 h at 30 °C, harvested by centrifugation at 8000 rpm for 2 min, washed twice with 0.9% sterile N-saline, and adjusted to a cell density about 1.0 × 10^8^ cells/mL to prepare the inoculum. All the experiments were performed in triplicate, and non-inoculated cultures served as controls.

### Microorganisms

*A. junii* LH-1-1 (Collection number: CGMCC 7.378, GenBank accession number: MK053916) was isolated from strawberry rhizosphere soil and could degrade 82.36% of deltamethrin (75 mg/L) at 30 °C in OMSM after incubation for 96 h. *Klebsiella pneumoniae* BPBA052 (Collection number: CGMCC 7.307, GenBank accession number: KY425746) was obtained from the soybean rhizosphere soil, and it could degrade 96.37% of 3-PBA (100 mg/L) within 72 h in mineral salt medium (Tang et al. 2019).

### Growth and degradation kinetics of degrading strains

Considering that a growth and degradation kinetics model can predict the status of degrading-strain and residue of pollutants in the future (Kamyabi et al. 2018; Kuppusamy et al. 2016), a logistic equation (Eq. 1) was used to describe the kinetics of bacterial growth, and a first-order-degradation kinetic model (Eq. 2) was used to describe the residue of substrates (deltamethrin or 3-PBA), where *X*_*0*_ represents the initial cell concentration (OD_600_), *X*_*m*_ represents the maximum cell concentration (OD_600_), *t* represents the culture time (h), and *μ*_*m*_ represents the maximum specific growth rate (h^−1^). *C*_*0*_ is the initial substrates concentration (mg/L), *C*_*t*_ is the substrates concentration at time *t* (mg/L), *t* is the degradation time (h), *k* is the degradation rate constant (h^−1^), and *t*_*1/2*_ is the half-life of substrates. Inoculum (5.0% v/v) was added to 100 mL OMSM containing 75 mg/L substrates in a 250 mL Erlenmeyer flask, OD_600_ and substrates concentration were measured at regular intervals.

1 $$ X = X_{0} e^{{u_{m} t}} /(1 - (X_{0} /X_{m} )(1 - e^{{u_{m} t}} )) $$

2 $$ C_{t} = C_{0} \times e^{ - kt} ,\;t_{1/2} = \ln 2/k $$

### Effect of inoculum proportion of strains LH-1-1 and BPBA052 on deltamethrin and 3-PBA degradation

The strains LH-1-1 and BPBA052 were incubated in LB liquid medium to prepare inoculum. With a total inoculum of 5% (v/v), strains LH-1-1 and BPBA052 were mixed at a proportion of 10:0, 7.5:2.5, 6.7:3.3, 5:5, 3.3:6.7, 2.5:7.5, and 0:10. Each inoculum was inoculated to 100 mL OMSM with 75 mg/L deltamethrin for 72 h to measure the residues of deltamethrin and 3-PBA, and non-inoculated cultures served as controls.

### Effect of inoculation sequences of strains LH-1-1 and BPBA052 on deltamethrin and 3-PBA degradation

Under the optimal inoculum proportion of strains LH-1-1 and BPBA052, three different inoculation sequences of strains LH-1-1 and BPBA052 were investigated. Sequence 1: LH-1-1 was first inoculated and cultured in OMSM for 24 h before inoculation of strain BPBA052. Sequence 2: strains LH-1-1 and BPBA052 were inoculated in the OMSM, simultaneously. Sequence 3: BPBA052 was first inoculated and cultured in OMSM for 24 h before inoculation of strain LH-1-1. Each sequence was inoculated to 100 mL OMSM with 75 mg/L deltamethrin at an interval of 12 h to measure the residues of deltamethrin and 3-PBA, and non-inoculated cultures served as controls.

### Metabolite characterization

Three sets of experiments (L, LP, C) were set up to investigate the degradation of deltamethrin and its metabolic products in the co-culture. In experiment L, 5% (v/v) inoculum of strain LH-1-1 was inoculated in 200 mL OMSM containing 75 mg/L of deltamethrin as a positive control. In experiment LP, under the optimal inoculation proportion and inoculation sequence, co-culture of strains LH-1-1 and BPBA052 (total inoculum of 5% (v/v)) were inoculated in OMSM. In experiment C, non-inoculated cultures served as negative control. The metabolites of deltamethrin at an interval of 12 h were extracted and identified by gas chromatography-mass spectrometry (GC–MS) as described by Tang et al. (2018a).

### Degradation of deltamethrin and its intermediate metabolite 3-PBA in soil environment

Soil samples were collected from top 0–10 cm of a field in Xihua University, Sichuan, China, which had never been contaminated by pyrethroid insecticides. The characteristics of the collected soil samples were as follows: total bacteria: 4.7 × 10^8^ CFU/g, pH: 6.38, organic matter: 18.40 g/kg, total nitrogen: 2.52 g/kg, total phosphorus: 0.43 g/kg, water content: 34.52% (w/w). After the soil was air-dried and sieved, deltamethrin was added to the soil to make the final concentration 20 mg/kg, and then kept overnight for further studies.

Both sterile and nonsterile soils were used to investigate the co-culture effect of strains LH-1-1 and BPBA052 on degradation of deltamethrin and its metabolite, 3-PBA. Each soil sample (L, LP, C) was prepared by using 500 g of nonsterile soil in 500 mL Erlenmeyer flask. In sample L, only strain LH-1-1 was added in the nonsterile soil. In sample LP, under the optimal inoculation proportion and inoculation sequence, co-culture of strains LH-1-1 and BPBA052 was added in the nonsterile soil. And non-inoculated, nonsterile soil served as sample C (control). The mentioned above experiments (L, LP, C) were also performed once again in sterile soil. All the experiments were performed in triplicate. The water content of these soil samples was maintained at 35.0 ± 5.0% and the temperature was maintained at 30 ± 5 °C. The soil samples were collected and the content of deltamethrin and 3-PBA was measured every day.

## Results

### Growth and degradation kinetics of the bacterial strains

**Fig. 1:**
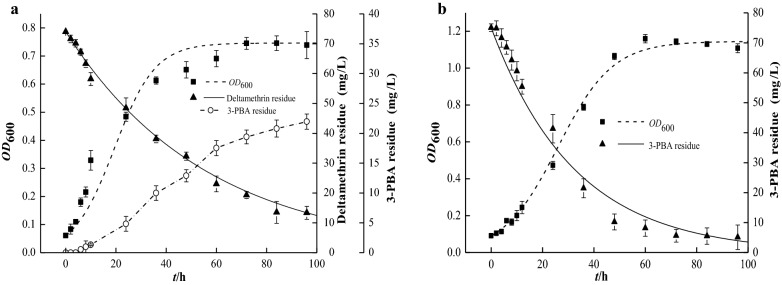
Growth and degradation curve of *A. junii* LH-1-1 **a** and *K. pneumoniae* BPBA052 **b**

As shown in Fig. 1, the experimental values of the biomass (OD_600_) of strains were nonlinearly fitted in the growth kinetics model. The OD_600_ of strain LH-1-1 rose from 0.06 to 0.74 and the growth kinetics parameters were *μ*_m_ = 0.12346 h^−1^, *X*_0_ = 0.06183, *X*_m_ = 0.74619 and substituting (Eq. 1) for the growth kinetics equation of strain LH-1-1: *X*_LH-1-1_ = 0.06183e^0.12346*t*^/(1–0.082861(1–e^0.12346t^)), R^2^ = 0.986. The results of growth kinetics of BPBA052 in OMSM containing 75 mg/L of 3-PBA were as follows: *X*_BPBA052_ = 0.0918e^0.09419*t*^/(1–0.080152(1–e^0.09419t^)), where *μ*_m_ = 0.09419 h^−1^, *X*_0_ = 0.0918, *X*_m_ = 1.14532, R^2^ = 0.985.

The first-order degradation kinetics model (Eq. 2) was used to nonlinearly fit the substrate residues in the degradation process. Within 96 h, more than 82% of the 75 mg/L deltamethrin initially added to the medium was degraded by LH-1-1 and a first-order degradation kinetics equation was obtained: *C*_LH-1-1_ = 74.02463e^−0.01805*t*^, *k *= 0.01805, *t*_1/2_ = 38.40 h, R^2^ = 0.996. The formation of 3-PBA was accompanied with the degradation of deltamethrin, reaching a maximum concentration of 22.40 mg/L in 96 h. It was noteworthy that the OD_600_ of strain LH-1-1 was 0.74, which was far lower than that of the strain BPBA052 (1.04). It may be because the toxic intermediate 3-PBA has antibacterial activity and inhibits microbial growth (Chen et al. 2011a). The first-order degradation kinetics equation of strain BPBA052 to degrade 3-PBA was as follows: *C*_BPBA052_ = 75.04785e^−0.03063*t*^, *k *= 0.03063, *t*_1/2_ = 22.63 h, R^2^ = 0.995, and it showed 93.34% of 3-PBA (75 mg/L) was degraded within 96 h in OMSM. The degradation rate was slightly slower in OMSM than in mineral salt medium, this inhibitory effect may be related to metabolism of additional carbon sources, which was also observed in the case of strain ZS-19 (Chen et al. 2015).

The R^2^ of the growth and degradation kinetics of strain LH-1-1 were 0.986 and 0.996, respectively. For the strain BPBA052 the values were 0.985 and 0.995, respectively. These results indicated that the two models can be quite well reflect the actual growth regularity of strains and the degradation regularity of substrates (deltamethrin or 3-PBA) by LH-1-1 or BPBA052, respectively.

### Effect of inoculum proportion on the degradation of deltamethrin and its metabolite 3-PBA

**Fig. 2:**
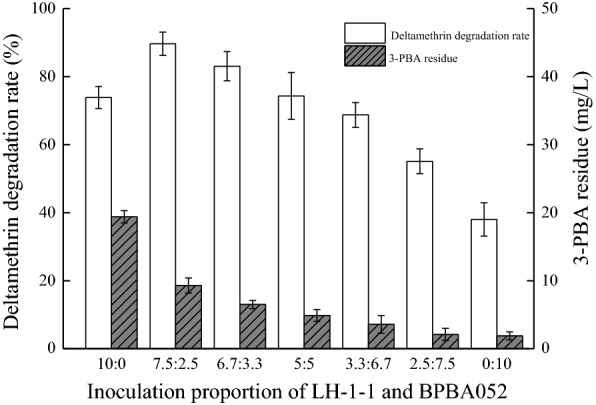
Deltamethrin degradation and 3-PBA content by using different inoculation proportions of strains LH-1-1 and BPBA052

Based on the degradation of different substrates by *A. junii* LH-1-1 and *K. pneumoniae* BPBA052, the co-culture of these two strains was used to improve the degradation of deltamethrin and decrease the content of metabolite 3-PBA during deltamethrin degradation process. As shown in Fig. 2, with the decrease in the amount of *A. junii* LH-1-1 inoculum and the increase in the amount of inoculum of *K. pneumoniae* BPBA052, the degradation rate of deltamethrin first increased and then decreased, while the content of 3-PBA gradually decreased within 72 h. When only strain LH-1-1 (10:0) was inoculated, the degradation rate of deltamethrin was 73.90% and a large amount of 3-PBA was accumulated (up to 19.41 mg/L), suggesting that the strain LH-1-1 could not effectively degrade 3-PBA, which could have also inhibited further degradation of deltamethrin. When only strain BPBA052 (0:10) was inoculated, the residue of 3-PBA was the least, but the degradation rate of deltamethrin was only 38.03%, because BPBA052 is an efficient 3-PBA degrading strain with poor deltamethrin-degrading ability. However, when the inoculation proportion of LH-1-1 and BPBA052 was 7.5:2.5, the degradation rate of deltamethrin reached the highest—89.67%, and only 9.30 mg/L 3-PBA remained at 72 h. Therefore, in our study the most appropriate proportion of strains LH-1-1: BPBA052 was 7.5:2.5, which used for subsequent studies.

### Effect of inoculation sequences on the degradation of deltamethrin and its metabolite 3-PBA

**Fig. 3:**
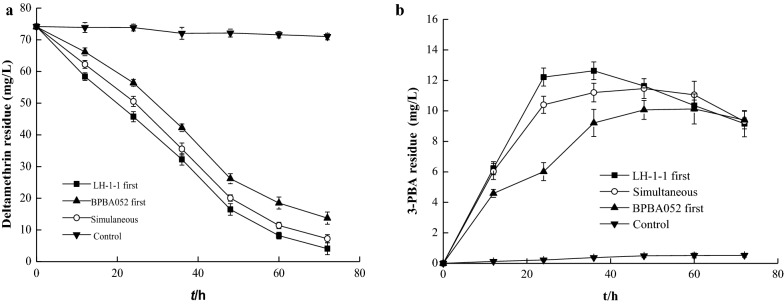
Effect of inoculation sequences of strains *A. junii* LH-1-1 and *K. pneumoniae* BPBA052 on degradation of deltamethrin **a** and its metabolite 3-PBA (**b**)

Based on the optimal inoculum proportion of the strains LH-1-1 and BPBA052, the optimal inoculation sequence of the strains for preparation of the co-culture was investigated. As shown in Fig. 3a, b, the residue of 3-PBA was not significantly different among the three different inoculation sequences within 72 h, but it is worth noting that there was a continuous degradation trend of 3-PBA, suggesting that 3-PBA might be completely metabolized with prolonged degradation time. But the degradation rate of deltamethrin by using sequences 1, 2, and 3 were 94.25%, 90.23%, and 81.45% within 72 h, respectively. This indicated that sequence 1 was the most efficient method, and the first-order degradation kinetics equation of the two strains by using sequence 1 to degrade deltamethrin was obtained: *C*_LH-1-1+BPBA052_ = 75.411e^−0.0282*t*^, *k *= 0.0282, *t*_1/2_ = 24.58 h, R^2^ = 0.974. The degradation rate of deltamethrin was 20.35% higher than that in monoculture *A. junii* LH-1-1, and the half-life was shortened from 38.40 h to 24.58 h. The results showed that deltamethrin could be efficiently co-degraded by *A. junii* LH-1-1 and *K. pneumoniae* BPBA052.

### Metabolic products and degradation pathway of deltamethrin by co-culture

**Fig. 4:**
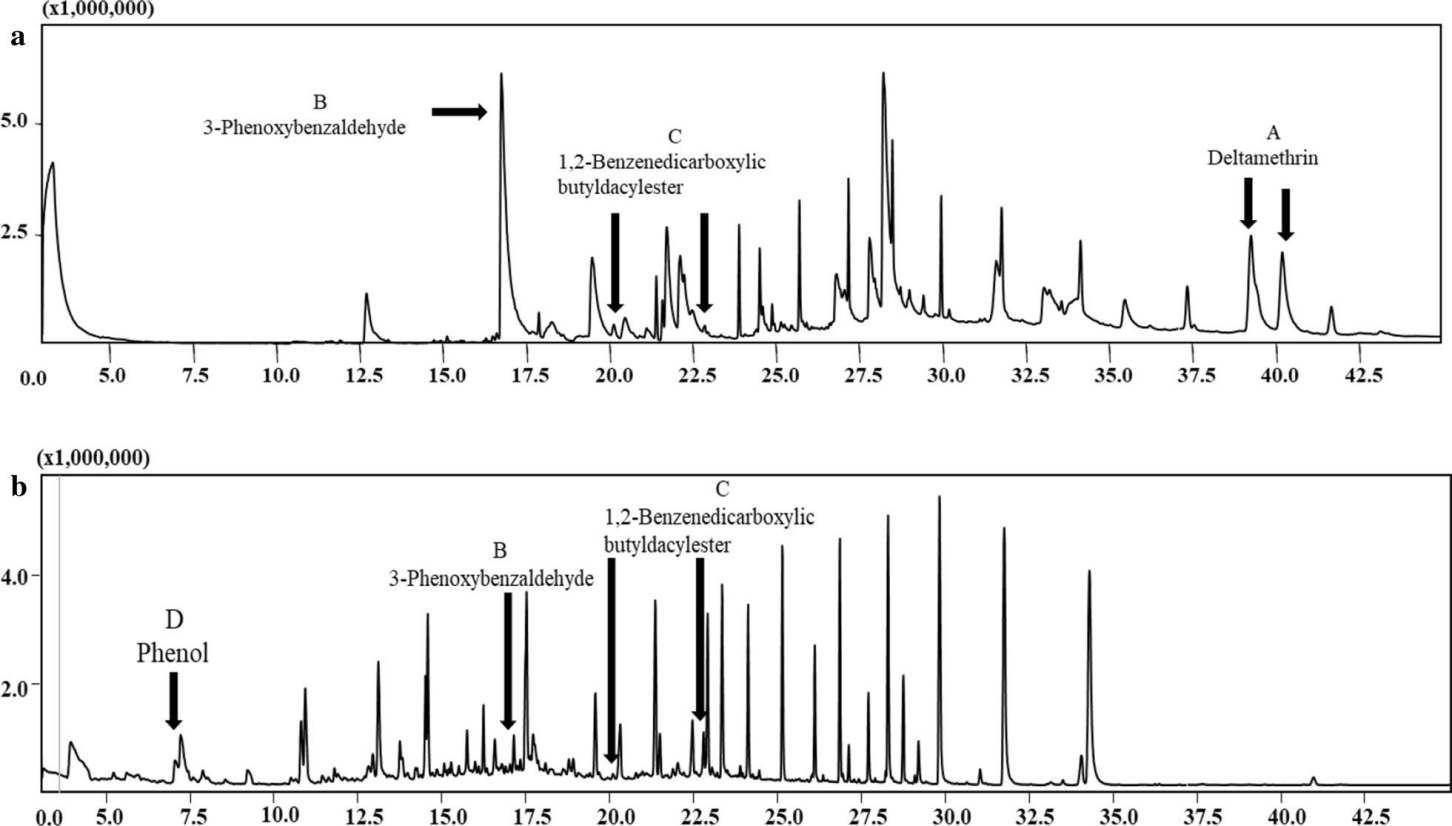
**a** GC–MS chromatogram of metabolites of deltamethrin obtained by degradation of deltamethrin in monoculture strain *A. junii* LH-1-1 within 72 h; and **b** co-culture strains *A. junii* LH-1-1 and *K. pneumoniae* BPBA052 within 72 h

**Fig. 5:**
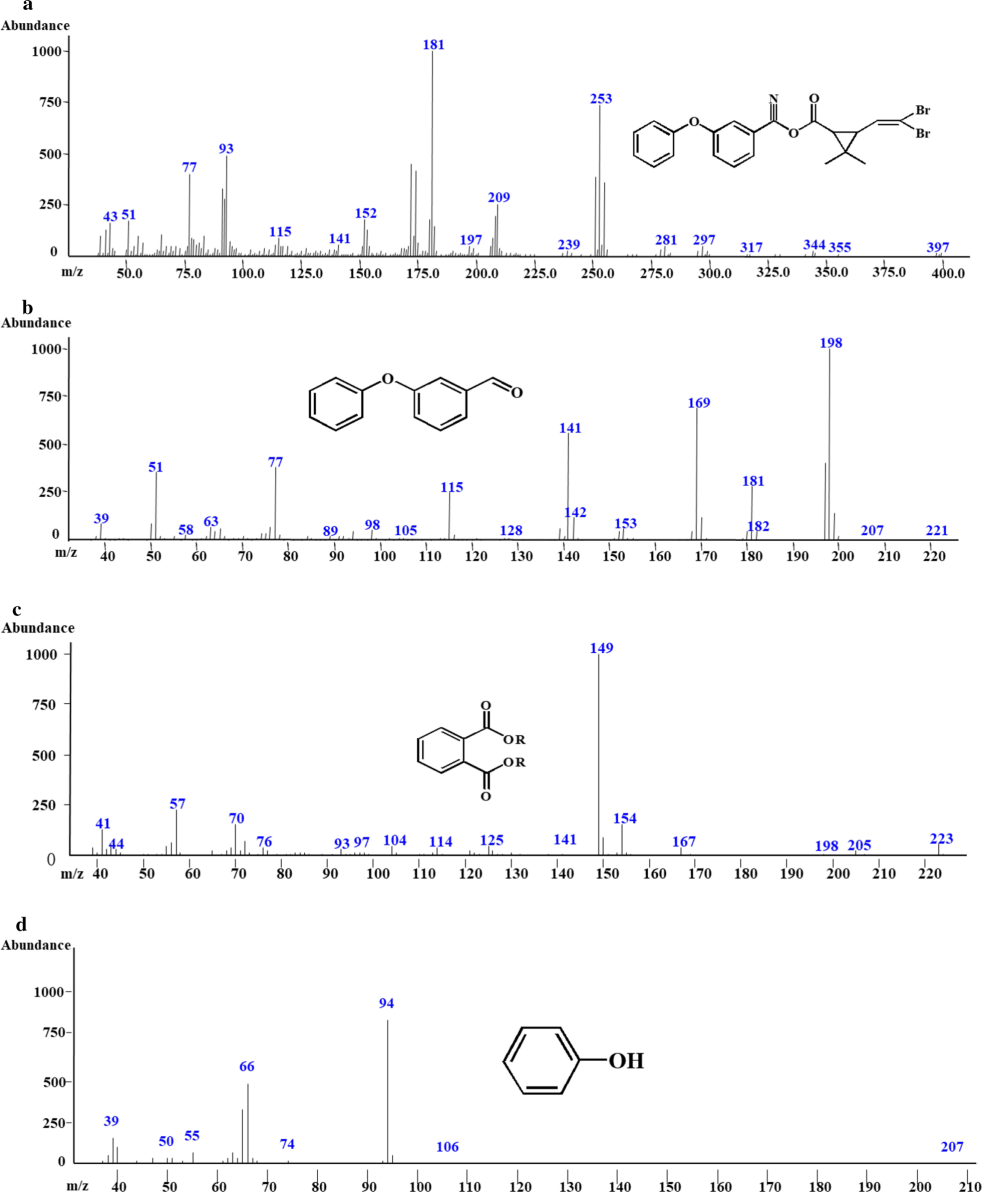
**a**–**c** m/z of compounds (A-C) in GC–MS from deltamethrin degradation by strain LH-1-1. **b**–**d** m/z of compounds (B-D) in GC–MS from deltamethrin degradation by co-culture of strains LH-1-1 and BPBA052

To reveal the degradation mechanism of deltamethrin by the co-culture of strains *A. junii* LH-1-1 and *K. pneumoniae* BPBA052, samples were detected and characterized by GC–MS (Fig. 4). And three main degradation products (Fig. 5 and Table 1) were identified during the degradation process with those of corresponding authentic standard compounds from the National Institute of Standards and Technology (NIST) library database.

**Table 1:** Chromatographic properties of metabolites of deltamethrin by strain LH-1-1, co-culture strains LH-1-1 and BPBA052

Compound	m/z	Retention time (min)	Chemical structural formula in NIST library	Similarity (%)	Name
A	505	39.267/41.683		93	Deltamethrin
B	198	16.792		95	3-Phenoxybenzaldehyde
C	278	20.167/22.275		96	1,2-benzenedicarboxylic butyl dacyl ester
D	94	6.525		95	Phenol

In experiment L (monoculture LH-1-1), three compounds were detected (Fig. 4a) within 72 h, compound A with significant peaks at retention times of 39.27 and 41.68 min showed major fragment ions at m/z 181 and 253, which was identified as deltamethrin. With the disappearance of compound A, two new compounds B and C were formed. According to their retention times and molecular ions after matching with the NIST library database, compound B was characterized as intermediate 3-phenoxybenzaldehyde, which was unstable and easily dehydrogenated into 3-PBA according to the research of Hu et al. (2018). Compound C was 1,2-benzenedicarboxylic butyl dacyl ester. It is noteworthy that, for the first time, compound C has been detected in the biodegradation pathway of deltamethrin.

**Fig. 6:**
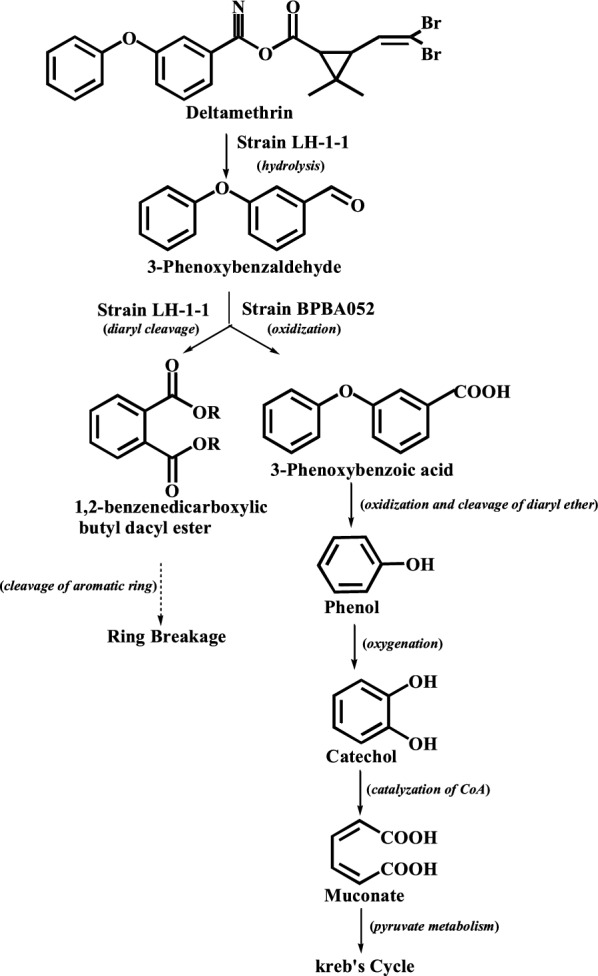
Proposed biodegradation pathway of deltamethrin and its metabolite 3-PBA by the co-culture of strains *A. junii* LH-1-1 and *K. pneumoniae* BPBA052

In experiment LP, the intermediate metabolites from co-culture degradation of deltamethrin were 3-phenoxybenzaldehyde (B), 1,2-benzenedicarboxylic butyl dacyl ester (C), and phenol (D) within 72 h. A new peak with retention time 6.525 min was observed and was identified as phenol, which was not detected in experiment L (monoculture strain LH-1-1). The formation of phenol may be due to the oxidization and cleavage of diaryl ether of 3-PBA by the strain BPBA052 (Tang et al. 2019). Along with metabolization, no persistent accumulative product was detected, the results showed that deltamethrin and its intermediate metabolites were degraded and completely mineralized by co-culture. Based on the chemical structures of deltamethrin and the identified metabolites, a microbial metabolic pathway of deltamethrin by co-culture of strains *A. junii* LH-1-1 and *K. pneumoniae* BPBA052 was proposed (Fig. 6). Firstly, deltamethrin (A) was hydrolyzed to yield 3-phenoxybenzaldehyde (B) and (1R, cis)-3-(2,2-dibromoethenyl)-2,2-dimethylcyclopropane carboxylic acid by strain LH-1-1. Secondly, a small amount of toxic intermediate 3-phenoxybenzaldehyde (B) was further metabolized with diaryl cleavage to form 1,2-benzenedicarboxylic butyl dacyl ester by strain LH-1-1, and through the cleavage of aromatic rings to achieve complete metabolism. While a large amount of transient 3-phenoxybenzaldehyde (B) was dehydrogenated into 3-PBA, which was rapidly degraded with oxidation and cleavage of diaryl ether to generate phenol (D) by strain BPBA052. Finally, phenol was converted to CO_2_ and H_2_O by the strain BPBA052 to achieve complete mineralization (Tang et al. 2019).

### Degradation of deltamethrin and its intermediate metabolite 3-PBA in soil environment

**Fig. 7:**
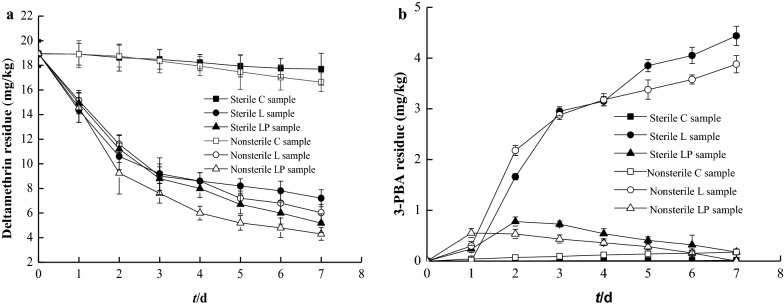
Deltamethrin residue **a** and 3-PBA residue **b** in the sterile soil and nonsterile soils by monoculture strain *A. junii* LH-1-1 (L sample), the co-culture *A. junii* LH-1-1 and *K. pneumoniae* BPBA052 (LP sample), and the uninoculated control (C sample)

The content of deltamethrin and 3-PBA in the sterile soil and nonsterile soils after degradation by monoculture strain *A. junii* LH-1-1 (L sample), co-culture strains *A. junii* LH-1-1 and *K. pneumoniae* BPBA052 (LP sample), and the control (C sample) are shown in Fig. 7. LP sample in nonsterile soil after incubation 7 d, showed 77.33% degradation of the 20 mg/kg deltamethrin initially added to the soil, and 3-PBA was not detected. While in L sample, the degradation of deltamethrin was only 68.10% and 3.878 mg/kg of 3-PBA was detected. These results showed that both in the medium or the natural soil environment, bioremediation by co-culture of strains LH-1-1 and BPBA052 was better than that in monoculture strain LH-1-1, as described previously by Liu et al. (2014). Similar results were also found in sterile soil, the degradation rate of deltamethrin in LP sample was 72.65%, which was 10.63% higher than that in L sample. And the content of 3-PBA was 0.18 mg/kg in LP sample, which was 4.26 mg/kg lower than that in L sample. These results further confirmed that co-culture strains LH-1-1 and BPBA052 has promising potential and advantages as bioremediation organisms in degrading deltamethrin and its intermediate metabolite 3-PBA in highly various environments.

## Discussion

Up to now, most studies effort has focused on screening and isolating highly effective pesticide-degrading strains from pesticide-contaminated environments, while ignoring how to improve the degradation of pesticides and the completely mineralized pathway of pesticides and their associated metabolites (Chen et al. 2011c; Deng et al. 2015). Here, when the inoculum proportion of the strains LH-1-1 and BPBA052 was 7.5:2.5, and LH-1-1 was inoculated 24 h before inoculation of strain BPBA052, the degradation of deltamethrin and 3-PBA concentration by co-culture of strains LH-1-1 and BPBA052 was 20.36% higher and 10.25 mg/L lesser than that in monoculture LH-1-1. This may be due to that strain LH-1-1 cannot quickly and completely degrade toxic intermediate 3-PBA, while BPBA052 possess high-efficient degradation ability in 3-PBA. Therefore, when a proper amount of BPBA052 was introduced, the toxicity inhibition of 3-PBA was quickly released in the degradation process, and the degradation of deltamethrin was further accelerated (Zhao et al. 2016). Liu et al. (2014) reported that the appropriate proportion of *Bacillus licheniformis* B-1 and *Sphingomonas* sp. SC-1 to degrade cypermethrin and its metabolite was 3.3:6.7, this signifies that the optimal inoculum proportion in case of different strains and substrates in a co-culture is different. But the inoculation sequence was confirmed the observations made by Liu et al.(2014), pesticide-degrading strains inoculated 24 h before inoculation of 3-PBA-degrading strains (sequence 1) was the most efficient method. And the degradation rate of deltamethrin and the residue of 3-PBA in our study were higher and lower, respectively, than that in co-culture strains B-1 and SC-1 (Liu et al. 2014). Thus, co-culture strains LH-1-1 and BPBA052 possess broad application potential for further bioremediation of PPs contaminated environment.

Although metabolic mechanism is the basis of application, reports on co-culture biodegradation pathway and mechanism are still rare (Chen et al. 2012b; Zhao et al. 2016). This research verified the degradation mechanism of deltamethrin and 3-PBA by co-culture of strains LH-1-1 and BPBA052 (Fig. 6). Firstly, deltamethrin (A) was hydrolyzed to yield 3-phenoxybenzaldehyde (B) and (1R, cis)-3-(2,2-dibromoethenyl)-2,2-dimethylcyclopropane carboxylic acid by strain LH-1-1. Previous evidences also indicated that esters are considered susceptible to degradation by microbes via hydrolysis, which is the main step in detoxification of PPs (Hu et al. 2018; Yang et al. 2018). This hydrolysis is similar to the initial step of β-cypermethrin degradation by *Bacillus licheniformis* B-1 reported by Zhao et al. (2016), wherein β-cypermethrin was hydrolyzed to 3-PBA. Regrettably, the further degradation of 3-PBA was not observed using strain B-1. Although Halden et al. (1999) and Topp and Akhtar (1991) reported that *Pseudomonas* sp. strains could utilize and transform 3-PBA, and Chen et al. (2012a) reported that *Bacillus* sp. DG-02 was capable of oxidizing 3-PBA to form 3-(2-hydroxyphenoxy) benzoic acid. But all these 3-PBA-degrading strains could not metabolize PPs. Fortunately, in our current study co-culture of strains LH-1-1 and BPBA052 not only achieved the detoxification of deltamethrin by ester bond hydrolysis, but further transformed its toxic metabolite (3-PBA) by cleavage of the diaryl bond, achieving completely mineralized pathway. That is a small amount of hydrolyzed 3-phenoxybenzaldehyde (B) was metabolized with diaryl cleavage to form 1,2-benzenedicarboxylic butyl dacyl ester by strain LH-1-1, and through the cleavage of aromatic rings to achieve complete metabolism. While a large amount of transient 3-phenoxybenzaldehyde (B) was rapidly degraded with oxidation and cleavage of diaryl ether by BPBA052 to generate phenol releasing the toxicity inhibition of 3-PBA and achieving completely mineralized pathway.

The potential of the co-culture strains LH-1-1 and BPBA052 to remediate deltamethrin- contaminated soil has been confirmed. Whether in sterilized soils or in unsterilized soils, the degradation rate of deltamethrin and 3-PBA concentration in co-cultured strains LH-1-1 and BPBA052 is much higher and lower than that in monoculture strain LH-1-1. And both in case of degradation by monoculture of strain LH-1-1 or co-culture of strains LH-1-1 and BPBA052, the degradation rate of deltamethrin in nonsterile soil was higher than that in sterile soil, and the content of 3-PBA in nonsterile soil was lower than that in sterile soil. These results confirmed the observations made by Chen et al. (2012a) and Zhan et al. (2018), suggesting that introduced strains and autochthonous microorganisms may have a synergistic effect in degrading xenobiotic compounds. Therefore, co-culture could be a promising strategy for remediation of environments contaminated with PPs.

In summary, the inoculum proportion and the inoculation sequence played an important role in effective degradation of deltamethrin in co-culture of strains LH-1-1 and BPBA052. And the co-culture degraded deltamethrin via a novel metabolic pathway with the formation of 3-phenoxybenzaldehyde by ester hydrolysis reaction by strain LH-1-1, which was further transformed by strain LH-1-1 and BPBA052 to form 1,2-benzenedicarboxylic butyl dacyl ester and phenol without any persistent accumulative product, indicating that the co-culture harbors a complete metabolic pathway for degradation and mineralization of deltamethrin. Simultaneously, the study revealed that use of co-culture is a potential and efficient strategy for bioremediation of environments contaminated with pyrethroid and their toxic intermediate metabolites.

## Data Availability

The corresponding author is responsible for providing all experimental data upon request.
